# Evaluating the Impact of Audits and Re-audits on Adherence to the Ottawa Knee Rules in a High-Volume UK Trauma Centre

**DOI:** 10.7759/cureus.87426

**Published:** 2025-07-07

**Authors:** Hamza Ahmed, Aima Gilani, Farah Mazhar, Marium Rizwan, Aarish Azeem, Abdur Rehman

**Affiliations:** 1 Trauma and Orthopaedics, Salford Royal NHS Foundation Trust, Manchester, GBR; 2 Spinal Surgery, Salford Royal NHS Foundation Trust, Manchester, GBR

**Keywords:** imaging, knee trauma, ottawa knee rules, quality improvement, radiation

## Abstract

Background

The Ottawa Knee Rules (OKR) are a validated clinical decision-making tool designed to minimise unnecessary radiographs in knee trauma, thereby reducing radiation exposure, optimising resource utilisation, and streamlining patient management. This study audits and re-audits the clinical compliance with OKR in radiography referrals by the Orthopaedic and Emergency Department (ED) teams at Salford Royal NHS Foundation Trust.

Methodology

A two-cycle retrospective audit was conducted, examining knee X-ray request forms submitted between October 2023 and March 2024 (Cycle 1) and March 2024 and September 2024 (Cycle 2). Each request was evaluated against the OKR criteria and cross-referenced with corresponding clinical notes. Target compliance was 100%. Educational interventions were implemented after Cycle 1 to improve adherence.

Results

In Cycle 1, only 41% of referrals documented at least one OKR criterion. This improved significantly to 91% in Cycle 2. Notable improvements were observed in specific OKR indicators, including documentation of inability to bear weight (14% to 57%) and isolated patellar tenderness (13% to 72%).

Conclusions

Educational interventions substantially improved OKR compliance among ED and Orthopaedic staff. Sustained efforts, including regular training and audits, are essential to maintain adherence, reduce unnecessary imaging, and ensure high-quality patient care.

## Introduction

Trauma-related knee injuries constitute a significant portion of emergency and orthopaedic assessments. Clinical decision-making tools such as the Ottawa Knee Rules (OKR) are essential to prevent unnecessary imaging while maintaining diagnostic accuracy. First developed in Ottawa, Canada, in 1995, OKR have demonstrated excellent sensitivity for identifying clinically significant fractures and have since been validated globally across diverse patient populations [1,2].

The OKR recommend radiographs only when certain post-injury clinical features are present, including advanced age, isolated patellar tenderness, fibular head tenderness, inability to flex the knee to 90 degrees, and inability to bear weight both immediately and in the emergency department for four steps. These rules are endorsed by the National Institute for Health and Care Excellence (NICE) and the Royal College of Radiologists (RCR), particularly within the iRefer Guidelines [3,4].

Despite widespread awareness, anecdotal observations at our institution suggested inconsistent documentation of OKR criteria in imaging requests. This audit aimed to quantify compliance levels, assess documentation adequacy, and implement targeted interventions to address gaps in knowledge and practice.

## Materials and methods

Objectives

The audit aimed to assess clinical compliance with the OKR in X-ray referrals for acute knee trauma and evaluate the adequacy of clinical information provided in electronic request forms. A further objective was to determine the effectiveness of an educational intervention in improving compliance, followed by a re-audit to assess subsequent changes.

Study design

A retrospective two-cycle clinical audit was conducted. All knee radiograph requests made via the Electronic Patient Record system for patients presenting with acute knee trauma were included. Clinical notes were accessed and cross-referenced to confirm documentation.

Exclusion criteria

Patients were excluded if they were under 18 years of age, had injuries more than seven days old, presented with altered consciousness, had paraplegia or multiple lower limb fractures, were pregnant, or had only superficial or soft tissue injuries.

Data collection

Cycle 1 was conducted from October 2023 to March 2024 and included 63 patients after applying the exclusion criteria. Cycle 2 was conducted from March 2024 to September 2024 and included 75 patients post-exclusion. Referrals were reviewed for documentation of any OKR criteria. Subgroup analysis was conducted according to referrer designation, which included senior house officers (SHOs), registrars, emergency nurse practitioners (ENPs), advanced nurse practitioners (ANPs), and triage nurses.

Interventions post-Cycle 1

Following the first cycle, several educational interventions were implemented. These included visual aids (posters and leaflets) in clinical areas, presentations during departmental meetings, informal and formal teaching sessions, and email communication summarising the OKR.

## Results

Referrer documentation compliance

The adequacy of documentation for each referrer group improved significantly from Cycle 1 to Cycle 2 (Tables 1, 2). Orthopaedic doctors and nurse practitioners both showed notable increases in appropriate documentation of OKR criteria.

**Table 1:** Referrer documentation compliance Cycle 1. SHO: senior house officer; ENP: emergency nurse practitioner; ANP: advanced nurse practitioner

Group	Adequate	% Adequate	Inadequate	% Inadequate
Orthopaedic SHOs/Regs	15	35.7%	27	64.3%
ENPs/ANPs/Triage	11	52.4%	10	47.6%

**Table 2:** Referrer documentation compliance Cycle 2. SHO: senior house officer; ENP: emergency nurse practitioner; ANP: advanced nurse practitioner

Group	Adequate	% Adequate	Inadequate	% Inadequate
Orthopaedic SHOs/Regs	38	92.9%	3	7.1%
ENPs/ANPs/Triage	30	88.2%	4	11.8%

Compliance with specific OKR criteria

Substantial improvement was noted across all five OKR criteria between the two cycles (Table 3).

**Table 3:** Criteria compliance.

Criterion	Cycle 1	Cycle 2
Age ≥55 years	52%	62%
Fibular head tenderness	3%	68%
Isolated patellar tenderness	13%	72%
Inability to flex to 90°	3%	65%
Inability to bear weight (four steps)	14%	57%
At least one criterion met	41%	91%

Pie chart: OKR compliance by referrer type (Cycle 2)

**Figure 1:**
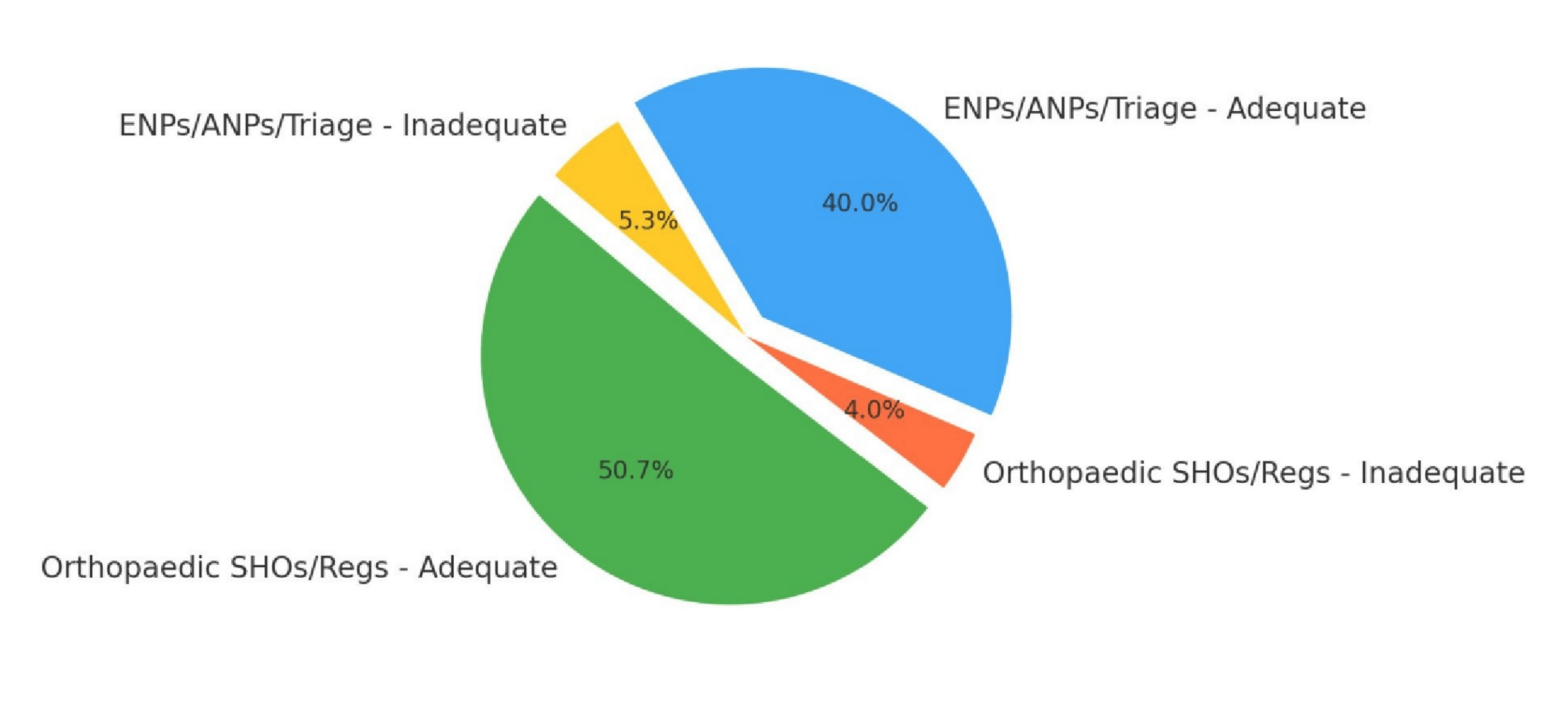
OKR documentation compliance by referrer type Cycle 2. OKR: Ottawa Knee Rules; SHO: senior house officer; ENP: emergency nurse practitioner; ANP: advanced nurse practitioner

Figure 1 shows the OKR documentation compliance of different referrer types in Cycle 2.

Chi-square test for association

A chi-square test revealed a statistically significant association between the audit cycle and adequacy of OKR documentation (p < 0.01), suggesting the educational interventions were effective in improving compliance.

Interpretation of audit cycles

In Cycle 1, only 41% of radiograph referrals documented at least one OKR criterion. Among these, fibular head tenderness and inability to flex to 90° were rarely noted (both 3%), indicating significant knowledge or documentation gaps. In contrast, age ≥55 years had the highest frequency (52%), likely due to its routine presence in demographic data.

Following the educational intervention, Cycle 2 showed a marked improvement to 91% compliance. The most dramatic improvements were seen in isolated patellar tenderness (from 13% to 72%), fibular head tenderness (from 3% to 68%), and the inability to flex (from 3% to 65%). These suggest the interventions effectively addressed the components of the OKR that were previously ignored or not paid attention to.

Referrer comparison

Orthopaedic doctors showed a considerable improvement from 35.7% to 92.9% in portraying adequate documentation. Nurse practitioners and triage staff also improved significantly (52.4% to 88.2%), indicating that the multi-professional format of training was successful.

## Discussion

This audit reinforces the clinical importance of the OKR and demonstrates that structured, low-cost educational interventions can yield significant improvements in adherence among both medical and nursing staff. The increase in documentation compliance from 41% to 91% suggests a substantial shift in the behaviour with regards to clinical practice, aligning with previous findings that multidisciplinary reinforcement enhances clinical decision tool adoption [5-7].

Several studies have emphasised the sensitivity of OKR (nearing 100%) for identifying fractures, with notable reductions in unnecessary radiographs when applied systematically [6]. Our findings throw light on those of Beutel et al. [1], who highlighted inconsistent documentation as a key barrier to OKR compliance. This was in particular for high-volume trauma centres. Similar to our audit, their intervention-driven study noted that education significantly increased both documentation rates and appropriate imaging decisions.

In addition to this, the improvement in fibular head tenderness and patellar tenderness documentation suggests that these lesser-known OKR elements are not something that people look at in routine, and hence, they require deliberate teaching. This mirrors findings by Cheung et al. [5], who identified these specific criteria as frequently overlooked in practice. Therefore, our results provide evidence that education must target not just the existence of OKR but the application of each component individually.

The greater improvement seen among orthopaedic junior doctors compared to ENPs and triage nurses may also reflect variation in prior exposure to clinical decision rules. Jalili and Gharebaghi [8] found that ongoing mentorship and involvement in audit processes reinforced longer-term compliance, a principle which could be applied to sustain our target goal of 100% compliance. This will lead to reductions in radiographs that are not needed, which is necessary to prevent radiation exposure to the patients [9].

Despite these improvements, 9% of Cycle 2 referrals still lacked adequate documentation. This may be explained by the rapid rotation of junior medical staff, variable IT proficiency, or insufficient reinforcement at the point of care. Integration of OKR prompts into electronic request systems, as recommended by NICE RCR’s iRefer guidelines [3,4], could further standardise practice and reduce reliance on individual memory.

Another noteworthy limitation is our reliance on documentation rather than real-time observation, which may have underestimated true clinical compliance. While this is a common limitation in retrospective audits [10], future studies might incorporate direct observation or simulation-based assessments to summarise the findings. Finally, the single-centre nature of our audit limits generalizability; however, the simplicity of the intervention and consistency of outcome improvements suggest that similar models could be replicated in comparable trauma units.

## Conclusions

This study demonstrates that simple, targeted educational interventions can significantly enhance clinical adherence to the OKR within acute trauma settings. By improving documentation and compliance among both medical and nursing staff, we observed a marked reduction in unnecessary radiographic investigations, and this was in line with evidence-based guidelines. The improvements observed across all five OKR criteria, particularly in the historically under-documented areas of fibular head tenderness and patellar tenderness, underscore the effectiveness of complex educational approaches. These results not only validate the OKR as a reliable tool for clinical decision-making but also highlight the critical role of continuous professional development, visual prompts, and regular audit cycles in ensuring we comply with the rules. We recommend that further steps include integration of OKR prompts into electronic requesting systems, mandatory induction teaching for new staff, and institutional policy reinforcement through local clinical governance. A standardised audit tool, such as the checklist provided in the appendix, may assist other departments in adopting similar quality improvement strategies. Ultimately, adopting consistent OKR adherence can streamline patient care, reduce healthcare costs, and enhance patient safety.

## Appendices

Checklist

**Table 4:** Checklist for evaluating the impact of audits and re-audits on adherence to the Ottawa Knee Rules (OKR) in a high-volume UK trauma centre. Instructions for Use: Complete the checklist for each knee trauma referral. Review both the imaging request and the corresponding clinical notes. Circle Yes/No for the OKR criteria and assess documentation quality. Use this tool for both initial audits and re-audits to monitor progress over time.

Patient identifier	Date of referral	Referrer role	Age ≥55 years	Isolated patellar tenderness	Fibular head tenderness	Inability to flex to 90°	Inability to bear weight (four steps)	At Least one OKR criterion met	Adequate documentation
			Yes/No	Yes/No	Yes/No	Yes/No	Yes/No	Yes/No	Yes/No
